# Supplementary material for: Controlling anammox speciation and biofilm attachment strategy using N-biotransformation intermediates and organic carbon levels
Source: Sci Rep. 2022 Dec 15;12:21720. doi: 10.1038/s41598-022-26069-2 (PMC9755228; doi:10.1038/s41598-022-26069-2)
Supplement: Supplementary file 1 — Supplementary Information. [file 41598_2022_26069_MOESM1_ESM.docx]

Controlling anammox speciation and biofilm attachment strategy using N-biotransformation intermediate and organic carbon levels

*Supplementary Information*

Yang Lu^1^*^#^, Gayathri Natarajan^1^*, Thi Quynh Ngoc Nguyen^1##^, Sara Swa Thi^1^, Krithika Arumugam^1^, Thomas William Seviour^1,2^**, Rohan B.H. Williams^3^, Stefan Wuertz^1,4^, Yingyu Law^1^

^1^ Singapore Centre for Environmental Life Sciences Engineering, Nanyang Technological University, Singapore 637551, Singapore.

^2^ Centre for Water Technology (WATEC) & Department of Biological and Chemical Engineering, Aarhus University, Universitetsbyen 36, 8000 Aarhus C, Denmark

^3^ Singapore Centre for Environmental Life Sciences Engineering, National University of Singapore, Singapore 119077, Singapore.

^4^ School of Civil and Environmental Engineering, Nanyang Technological University, Singapore. 639798, Singapore.

* Y. Lu & G. Natarajan contributed equally to this paper. Author order was determined alphabetically.

** Correspondence to: T.W. Seviour (twseviour@bce.au.dk)

^#^ Current affiliation: The Australian Centre for Ecogenomics, School of Chemistry and Molecular Biosciences, University of Queensland, St Lucia, Queensland, Australia 4072

^##^ Current affiliation: Agency for Science, Technology and Research, Singapore 138632, Singapore.

**Supplementary Information**

Number of pages: 6

Number of tables: 3

Number of figures: 3

**Paired end Illumina Miseq 16S rRNA amplicon sequencing data analysis**

The raw paired reads were processed by an in-house script provided by DNAsense including: High quality reads, generated after trimming and quality filtering by Trimmomatic (V0.32, Bolger et al., 2014), were subjected to be merged by flash ^1^. USEARCH ^2^ was then used to remove Phix contamination by command usearch global, dereplicate the reads by command fastq_filter, cluster dereplicated reads to operational taxonomy units (OTUs) at 97% similarity by command cluster_OTUs and map the contamination free reads to OTUs by command usearch_global. Taxonomy annotation was assigned to OTUs by QIIME (v1.9.0) (Caporaso et al., 2010) using SILVA database (132 release). Alpha diversities including Chao1 and Simpson index of diversity were calculated by QIIME, rarefied to 16,000 sequences per sample and 10 times iterations on samples collected from day 149 to 255 (11 samples) of R1, day 156 to 265 (14 samples) of R2 and day 105-274 (8 samples) of R3. ANOSIM analysis were carried out on microbial community data between reactors, and between period with different N load of the same reactor by ‘anosim’ ^3^ of package vegan in R (v3.6.2) ^4^.

***Candidatus* Brocadia specific primer and probe design**

A *Candidatus* Brocadia specific forward primer was modified from *Planctomycetales*-specific primer Pla46 ^5^ with correction of mismatch to a full length 16S rRNA gene identified in the *Ca.* Brocadia draft genome recovered from R1 ^6^. The designed *Ca.* Brocadia specific primer was used with 1492R ^7^ to amplify 1509 bp of the 16S rRNA gene. PCR was carried out with the following conditions: 5 min initial denaturation at 94 ^o^C, 25 cycles of 1 min at 94 ^o^C, primer annealing for 1 min at 50 ^o^C, 70 sec at 72 ^o^C and 4 min at 72 ^o^C as final extension. Species specific oligonucleotide probes were designed targeting *Ca.* B. caroliniensis (*Bca183*) and *Ca.* B. sinica (*Bsi630*), respectively, using the probe design tool of the ARB software package ^8^ according to Hugenholtz et al. ^9^. Based on comparative analysis of all sequences in the Silva database (v132 release), the program selected regions within the target sequences recovered from the clone library (as described below) and draft genome ^6^, which allowed the design of probes specific for all or part of *Ca.* B. caroliniensis and *Ca.* B. sinica. Mismatches to non-target sequences were centralized by adjusting the design parameters used (where possible) to minimise the risk of unspecific binding. Probe sequences were subsequently confirmed for specificity using BLAST. The stringency conditions for FISH probes were assessed with a range of formamide concentrations (5%, 10%, 20%, 30%, 35% and 55%) as described in the FISH section under materials and methods in the main manuscript. Samples collected from a laboratory reactor without AnAOB were used as a negative control in both PCR and FISH.

**Clone library construction**

16S rRNA genes were amplified from total DNA using the designed forward primer (5’-RCATGCAAGTCGAACGWG-3’) and reverse primer 1492R (5’-ACCTTGTTACGACTT-3’). A clone library was generated with the TOPO TA cloning kit (Invitrogen, Carlsbad, CA) with pCR™4-TOPO® TA vector and One shot Chemically Competent cell TOP10, following the manufacturer’s manual. For each clone library, 40 colonies were picked and purified for Sanger sequencing with primer M13 forward and reverse respectively. Contigs were generated by DNA baser assembler (v5, Heracle Software). Chimeric artifacts were checked by decipher online tool (<http://decipher.cee.wisc.edu/FindChimeras.html>) and removed.

**Phylogenetic tree construction**

The chimera-free contigs, from clone library, along with representative sequences of dominant OTUs, from microbial community profiling, were aligned by SINA online tool (https://www.arb-silva.de/) and imported into SILVA reference tree (v132 release) by parsimony insertion tool in ARB (v6.0.4) ^8^. Sequence associated information was constructed based on OTUs with filtering by maximum frequency and used as filter for insertion. The closest neighbour sequences were selected along with inserted contigs and dominant OTUs for phylogenetic tree construction by distance matrix + neighbour joining methods with 1000 bootstrapping.

**Table S1.** Composition of the primary effluent augmented with nitrite (values are mean and standard deviation of 35 samples measured throughout the experiment).

| **Compound** | **Concentration** |
| --- | --- |
| Total Kjeldahl nitrogen (mg N/L) | 39.4 ± 11.7 |
| Total Phosphorus (mg P/L) | 7.1 ± 1.1 |
| Ammonium (mg N/L) | 35.8 ± 4.4 |
| Nitrite (mg N/L) | 70.1 ± 10.3 |
| Phosphate (mg P/L) | 4.1 ± 1.7 |
| Total COD (mg/L) | 260 ± 40 |
| Total Alkalinity (mg/L CaCO_3_) | 160 ± 25 |
| Acetate (mg/L) | 27.5 ± 13.1 |
| Propionate (mg/L) | 4.2 ± 2.5 |
| Butyrate (mg/L) | 0.3 ± 0.6 |

**Table S2.** Particle size analysis of suspended biomass samples from Reactor 1 (R1), Reactor 2 (R2) and Reactor 3(R3) collected during Phase III of reactor operation. Measurements were done in triplicate for each sample.

|  | |  |  |  |  |  |  |
| --- | --- | --- | --- | --- | --- | --- | --- |
|  | **25%D**  **(µm)** | | **50%D**  **(µm)** | **75%D (µm)** | **Median**  **(µm)** | **Mean V (µm)** | **Std Dev** |
| **R1** | | 909.8 | 1098.3 | 1322.2 | 1098.3 | 1089.4 | 0.1 |
|  | |  |  |  |  |  |  |
| **R2** | | 1370.4 | 1556.9 | 1739.8 | 1556.9 | 1527.8 | 0.1 |
|  | |  |  |  |  |  |  |
| **R3** | | 255.5 | 328.9 | 402.2 | 328.9 | 310.3 | 0.1 |

**Table S3.** Average diversity index and mean standard deviation (SD) were calculated based on samples collected after the nitrogen load increased in R1 (11 samples) and R2 (14 samples) and when Anammox bacteria were detected in R3 (8 samples), for each reactor in terms of total, Anammox and non-Anammox microbial community respectively.

|  | Total Community | | Anammox community | | Non-Anammox community | |
| --- | --- | --- | --- | --- | --- | --- |
| Reactor | Chao1±SD | Simpson*±SD | Chao1±SD | Simpson±SD | Chao1±SD | Simpson±SD |
| R1 | 233±31 | 0.11±0.02 | 19±3 | 0.23±0.05 | 283±42 | 0.10±0.04 |
| R2 | 249±32 | 0.09±0.03 | 21±5 | 0.22±0.02 | 293±20 | 0.08±0.03 |
| R3 | 614±42 | 0.04±0.01 | 30±7 | 0.75±0.17 | 585±45 | 0.04±0.01 |

*Simpson index of diversity is calculated as 1-D as default settings by QIIME.


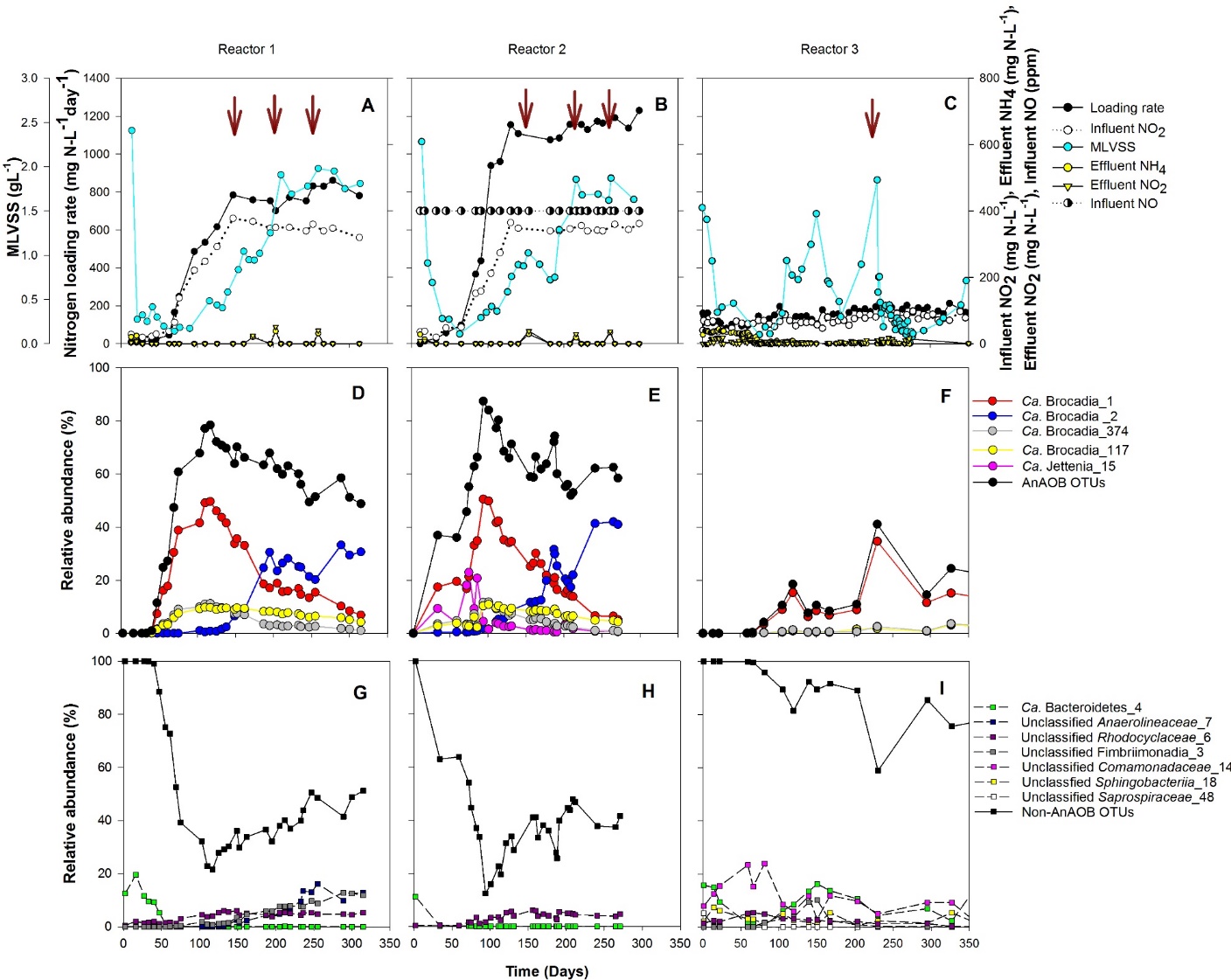


**Figure S1.** Start-up and enrichment of Anammox reactor from activated sludge fed with (A, D, G) synthetic waste water with ammonium and nitrite-Reactor 1, (B, E, H) synthetic waste water with ammonium, nitrite and continuous supply of nitric oxide-Reactor 2, and (C, F, I) primary effluent supplemented with nitrite-Reactor 3. Mixed liquid volatile suspended solids (MLVSS), Influent nitrite load and nitrogen loading rates of each enrichment condition are shown in A, B and C with the corresponding relative abundance of annotated AnAOB taxa (with minimum detectable relative abundance of above 10% at any given time point) from suspended biomass samples in D, E and F and corresponding relative abundance of annotated dominant non-ANAOB in G, I and H based on 16s rRNA amplicon sequencing. Brown arrows in the top panel denote the time points at which Anammox biofilm was scraped from the wall of the reactor into suspension.


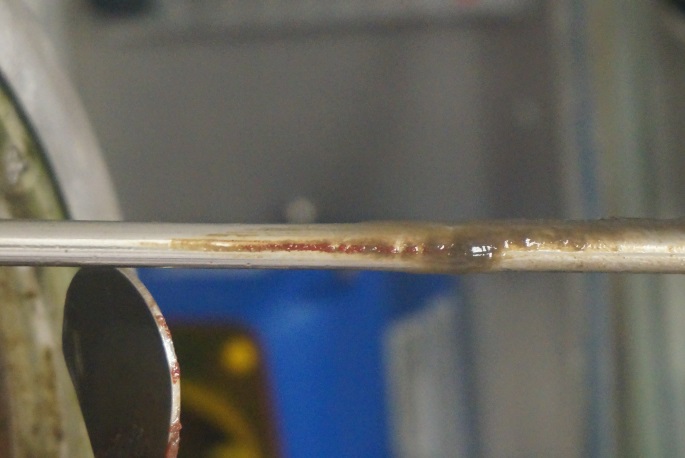


**B**


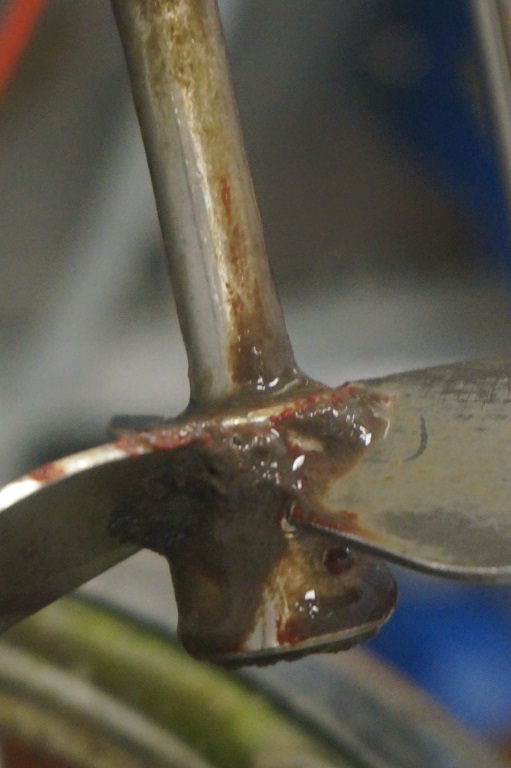


**A**


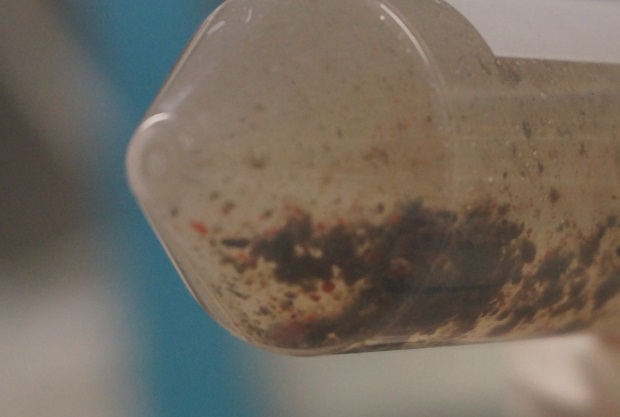


**C**

**Figure S2.** Images of biofilm formation on the surface of the stirrer (A) and (B), and the (C) sludge in suspension after removal of biofilm from reactor wall for Reactor 3 enriched with primary effluent augmented with nitrite.


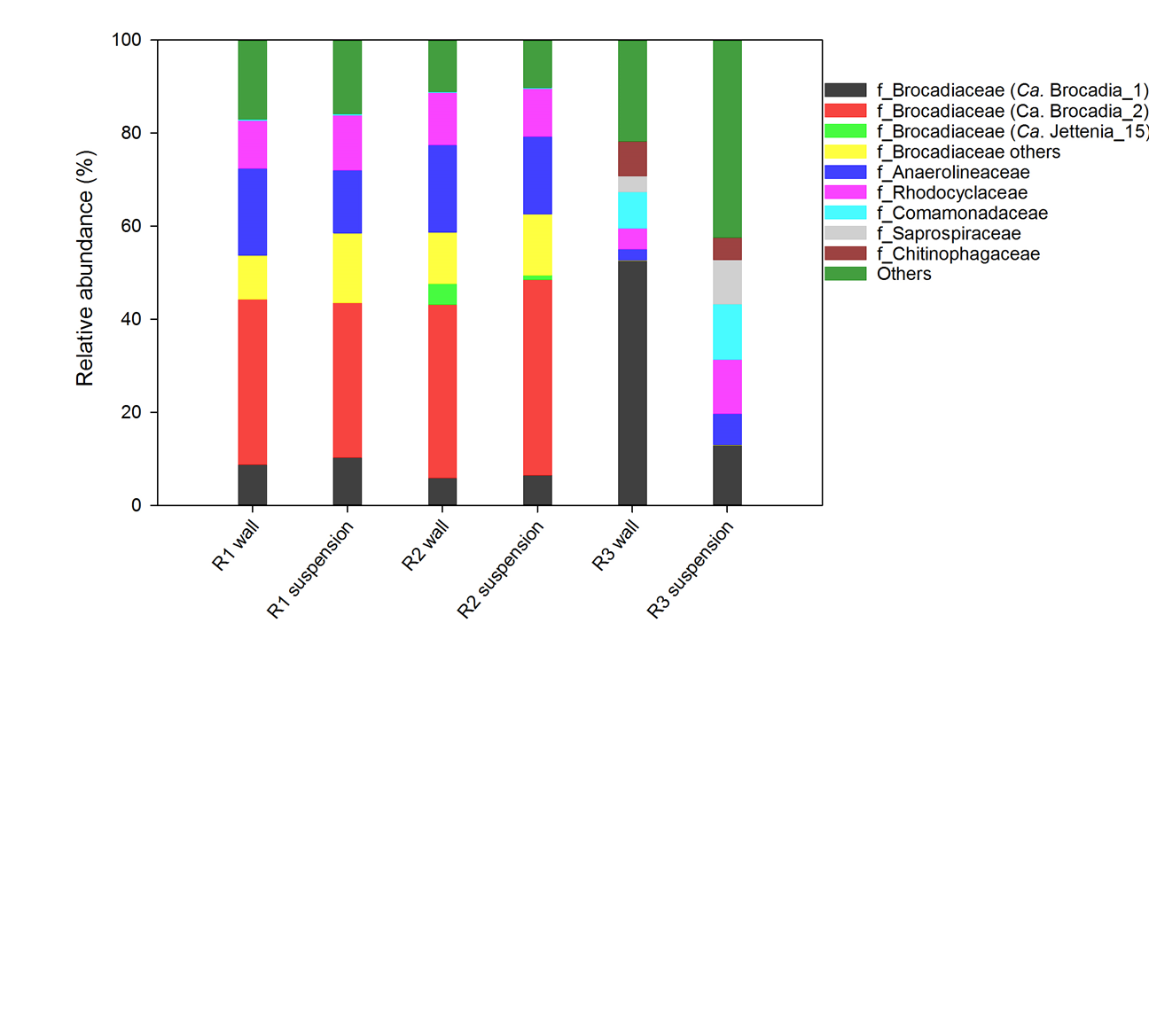


**Figure S3.** Relative abundance of OTUs detected in samples collected from wall and mixed liquor suspension in reactors R1 (day 289), R2 (day 265) and R3 (day 266). *Ca.* Brocadia_1 (*Ca.* Brocadia caroliniensis), *Ca.* Brocadia_2 (*Ca.* Brocadia sinica) and *Ca.* Jettenia are represented at the OTUs level and others are summed to family level.

**References:**

1 Magoč, T. & Salzberg, S. L. FLASH: fast length adjustment of short reads to improve genome assemblies. *Bioinform.* **27**, 2957-2963 (2011).

2 Edgar, R. C. Search and clustering orders of magnitude faster than BLAST. *Bioinform.* **26**, 2460-2461 (2010).

3 Oksanen, J. *et al.* Vegan: Community Ecology Package. R package version 2.5-6. [*https://CRAN.R-project.org/package=vegan*](https://CRAN.R-project.org/package=vegan) (2019).

4 Team, R. C. R: A language and environment for statistical computing. R Foundation for Statistical Computing, Vienna, Austria. URL

[*https://www.R-project.org/*](https://www.R-project.org/) (2019).

5 Neef, A., Amann, R., Schlesner, H. & Schleifer, K.-H. Monitoring a widespread bacterial group: in situ detection of planctomycetes with 16S rRNA-targeted probes. *Microbiology* **144**, 3257-3266 (1998).

6 Liu, X. *et al.* Draft genome sequence of a "*Candidatus* Brocadia" bacterium enriched from activated sludge collected in a tropical climate. *Genome Announc* **6**, e00406-00418 (2018).

7 Lane, D. 16S/23S rRNA sequencing. *Nucleic acid techniques in bacterial systematics*, 115-175 (1991).

8 Ludwig, W. *et al.* ARB: a software environment for sequence data. *Nucleic Acids Res.* **32**, 1363-1371 (2004).

9 Hugenholtz, P., Tyson, G. W. & Blackall, L. L. in *Gene probes* 29-42 (Springer, 2002).
